# Visible light crosslinkable human hair keratin hydrogels

**DOI:** 10.1002/btm2.10077

**Published:** 2018-01-19

**Authors:** Kan Yue, Yanhui Liu, Batzaya Byambaa, Vaishali Singh, Wanjun Liu, Xiuyu Li, Yunxia Sun, Yu Shrike Zhang, Ali Tamayol, Peihua Zhang, Kee Woei Ng, Nasim Annabi, Ali Khademhosseini

**Affiliations:** ^1^ Div. of Engineering in Medicine, Dept. of Medicine, Biomaterials Innovation Research Center Brigham and Women's Hospital, Harvard Medical School Cambridge MA 02139; ^2^ Harvard‐MIT Division of Health Sciences and Technology Massachusetts Institute of Technology Cambridge MA 02139; ^3^ College of Textiles, Donghua University Shanghai 201620 China; ^4^ School of Materials Science and Engineering Nanyang Technological University, N4.1, 50 Nanyang Avenue Singapore 639798 Singapore; ^5^ Research Center for Analysis and Measurement Hebei Normal University Shijiazhuang 050024 Hebei China; ^6^ Dept. of Chemistry and Key Laboratory of Biomedical Polymers, Ministry of Education Wuhan University Wuhan 430072 China; ^7^ Wyss Institute for Biologically Inspired Engineering, Harvard University Boston MA 02115; ^8^ Dept. of Chemical Engineering Northeastern University Boston MA 02115; ^9^ Dept. of Bioindustrial Technologies College of Animal Bioscience and Technology, Konkuk University, Hwayang‐dong Gwangjin‐gu Seoul 143‐701 Republic of Korea; ^10^ Nanotechnology Center King Abdulaziz University Jeddah 21569 Saudi Arabia

**Keywords:** compounds/materials, regenerative medicine, tissue engineering

## Abstract

Keratins extracted from human hair have emerged as a promising biomaterial for various biomedical applications, partly due to their wide availability, low cost, minimal immune response, and the potential to engineer autologous tissue constructs. However, the fabrication of keratin‐based scaffolds typically relies on limited crosslinking mechanisms, such as via physical interactions or disulfide bond formation, which are time‐consuming and result in relatively poor mechanical strength and stability. Here, we report the preparation of photocrosslinkable keratin‐polyethylene glycol (PEG) hydrogels via the thiol‐norbornene “click” reaction, which can be formed within one minute upon irradiation of visible light. The resulting keratin‐PEG hydrogels showed highly tunable mechanical properties of up to 45 kPa in compressive modulus, and long‐term stability in buffer solutions and cell culture media. These keratin‐based hydrogels were tested as cell culture substrates in both two‐dimensional surface seeding and three‐dimensional cell encapsulation, demonstrating excellent cytocompatibility to support the attachment, spreading, and proliferation of fibroblast cells. Moreover, the photocrosslinking mechanism makes keratin‐based hydrogel suitable for various microfabrication techniques, such as micropatterning and wet spinning, to fabricate cell‐laden tissue constructs with different architectures. We believe that the unique features of this photocrosslinkable human hair keratin hydrogel promise new opportunities for their future biomedical applications.

## 1 INTRODUCTION

Protein‐based biomaterials, such as those based on silk fibroin,^1^, ^2^ collagen,^3^, ^4^ and elastin,^5^ have been extensively studied to fabricate tissue engineered scaffolds. In general, protein‐based biomaterials demonstrate excellent biocompatibility and biodegradability via enzymatic or hydrolytic pathways. They also possess inherent bioactivities due to the presence of functional domains or peptide motifs along their backbones. However, widespread clinical applications of protein‐based hydrogels are limited partially by challenges toward large‐scale availability at low costs. Synthetic or recombinant peptide oligomers or proteins are still relatively expensive to use as scaffold materials.^6^ Conversely, despite certain successes, proteins extracted from animal sources are associated with potential safety concerns, including possible disease transmission or severe immune responses arising from exogenous proteins after implantation into human body.

Keratins are a class of proteins with variable compositions that widely exist in different epithelial tissues.^7^, ^8^ As one of the most important structural proteins, keratins assemble into filaments to protect epithelial cells, and also are the major component of hard protective tissues, such as hair, nail, and even horns in some animals. The dynamic breakage and formation of disulfide bonds between cysteine residues in keratins is the most significant process for the hierarchical assembly of the protein molecules.^8^ It has been long known that soluble keratins can be extracted from different sources, such as wool, feather, human hair, and so on, by chemical treatments to partially break the disulfide bonds.^9^ The resulting proteins can form porous scaffolds including cast films,^10^, ^11^, ^12^ sponges,^13^, ^14^, ^15^ and hydrogels,^16^, ^17^, ^18^ which have been used in various drug delivery^19^, ^20^ and tissue engineering applications, such as to reconstruct ocular surface,^11^, ^12^ to treat cardiac dysfunction,^21^ and to repair nerve defects.^22^, ^23^ However, most of previously reported keratin scaffolds rely on network formation via physical interactions and/or spontaneous formation of new disulfide bonds.^18^, ^24^ This can be a slow process and thus largely limit the encapsulation of cells within keratin‐based scaffolds. Moreover, the resulting network structures are typically not well defined, and suffer from weak mechanical strength and poor stability, thus limiting their applications in the biomedical field.

Therefore, there is an unmet need to engineer keratin‐based biomaterials that would benefit from an alternative crosslinking mechanism to form organized networks. Among the various crosslinking mechanisms reported to date, photoinitiated crosslinking has several important advantages, including tunable physical properties, rapid reaction kinetics, and precise spatial and temporal control to form patterned architectures.^25^, ^26^ Meanwhile, photoinitiators that can be activated by visible light instead of UV light are preferred, since the use of UV light in biomedical applications might cause possible cell and tissue damages.^27^ Taking advantage of the rich content of cysteine residues in keratins, we propose to apply a well‐documented “click”‐type reaction between thiol and norbornene groups, which can be viewed as a special subcategory of the thiol‐ene “click” reaction,^28^, ^29^ to form chemically crosslinked hydrogel networks, as first demonstrated in preparing synthetic extracellular matrix by Fairbanks and colleagues.^30^ Since then, this step‐growth photopolymerization technique based on the thiol‐norbornene reaction has found increasing applications in the field of biomaterials,^27^, ^31^, ^32^, ^33^ because it requires lower free radical concentrations that reduce toxicity, and possesses click‐type features to generate crosslinking sites in a well‐defined stoichiometric manner.

In this study, we present the preparation and characterization of keratin‐based hydrogels that can be photocrosslinked by using visible light. The crosslinking reaction took place between the free thiol groups on keratins and the norbornene functional groups on a four‐armed polyethylene glycol (PEG) linker (PEG‐4Nor).^32^, ^33^ A visible light photoinitiator Eosin Y was used to initiate the rapid polymerization to result in hydrogels within one minute under physiological conditions.^27^ We expect that by changing the concentrations of prepolymers and Eosin Y, as well as light irradiation time, physical properties of the resulting keratin‐PEG hydrogels can be readily tuned. In vitro studies were performed to show that the keratin hydrogels can support the attachment, spreading, and proliferation of NIH/3T3 fibroblasts in both two‐dimensional (2D) and three‐dimensional (3D) environments. Moreover, the photocrosslinking mechanism also allows microfabrication of keratin hydrogels using micropatterning with a photomask^34^, ^35^ and wet spinning^36^, ^37^ techniques. To our knowledge, there is no previous report on visible light crosslinkable, microfabricated keratin hydrogels. The development of such a system might provide a promising alternative strategy toward the preparation of a new class of human protein‐based hydrogel.

## 2 MATERIALS AND METHODS

### 2.1 Keratin extraction from human hair

Extraction and purification of keratin samples from human hair was performed following the previously described procedures.^15^, ^18^ Briefly, randomized human hair obtained from salons in Singapore were washed to remove contaminations, delipidized by a mixture of chloroform and methanol (v/v = 2/1), and cut into sections of less than 1 cm length. The pretreated hair sample (50 g) was then immersed in 1 L 0.125 M sodium sulfide solution (pH 10 − 13.5) at 40 °C for 2 hr. The resulting mixture was filtered through a filter paper, dialyzed against deionized water for 3 days, and lyophilized to afford a brown powder. The typical yield of this procedure was 20–30% by mass.

### 2.2 Synthesis of reduced human hair keratin (keratin‐SH)

Lyophilized keratin (500 mg) was dissolved in 10 mL deionized water at room temperature, followed by the addition of 200 mg 1,4‐dithiothreitol (DTT, 97%, Sigma‐Aldrich). The resulting mixture was stirred at room temperature overnight to break the disulfide bonds to enrich for free thiol groups. After that, the solution was purified using centrifugal filter units (Amicon Ultra‐15 with Ultracel‐3k membrane, MWCO 3000) by spinning at 4000g for 30 min. The concentrated protein solution in the filter was diluted to 15 mL with degassed deionized water, and then spinning for another 30 min at 4000g. The purification was repeated for five cycles to fully remove the excess DTT, which is crucial since DTT also contains thiol groups. The purified protein solution was neutralized using 0.01 M NaOH solution and then lyophilized to afford Keratin‐SH as a brown foam. The typical yield of this procedure was 70–80% by mass. Free thiol content in the pristine extracted keratin and the reduced Keratin‐SH samples were measured by the Ellman's assay (Thermo Scientific) following the instructions.^19^

### 2.3 Synthesis of the PEG‐4Nor

The PEG‐4Nor linker was prepared from four‐armed PEG precursor (MW ∼10 k with a pentaerythritol core, JenKem Tech, 95%) following the previously reported procedure.^32^ The degree of norbornene functionalization was determined to be >93% based on Proton nuclear magnetic resonance (^1^H NMR) data.

### 2.4 Preparation of visible light crosslinkable keratin‐PEG hydrogels

Keratin‐SH and PEG‐4Nor were dissolved in deionized water at different total concentrations (10–20% [w/v]) to prepare the prepolymer solutions. Both Keratin‐SH and PEG‐4Nor could fully dissolve at this concentration range, and the resulting mixed solution was clear. The ratio of thiol groups and norbornene groups was fixed to the stoichiometric ratio, which corresponds to a mass ratio of Keratin‐SH/PEG‐4Nor to be 1:1.3. To these solutions, 2′,4′,5′,7′‐tetrabromofluorescein (Eosin Y, Sigma‐Aldrich, 99%) disodium salt was added at desired concentrations (0.06 or 0.6 mM) as the photoinitiator. Photocrosslinked Keratin‐PEG hydrogels were formed by pipetting the prepolymer solution into a polydimethylsiloxane (PDMS) mold, which was covered with a glass slide and then exposed to visible light at an intensity of 70 000 Lux using a dual‐fiber optical microscope illuminator (AmScope).

### 2.5 Swelling ratio measurements

Swelling ratios of different keratin‐PEG hydrogels were measured following previously reported procedures.^6^, ^38^ To prepare the samples for swelling ratio tests, 100 µL prepolymer solution was pipetted into a PDMS mold with a diameter of 8 mm and a depth of 2 mm. The solution was then covered with a glass coverslip and irradiated by visible light for 5 min. After that, the formed hydrogels were removed from the mold and the coverslip, soaked in phosphate buffered saline (PBS, Life Technologies) and incubated at 37 °C for 24 hr to reach the equilibrium swelling state. After wiping off the excess liquid, the swollen hydrogel samples were weighed to record the equilibrium swelling mass (*W*_wet_). The hydrogel samples were then lyophilized to record their dry masses (*W*_dry_). The swelling ratio of was calculated according to the equation: (*W*_wet_–*W*_dry_)/*W*_dry_ × 100%. All the tests were performed using at least three samples for each hydrogel formulation.

### 2.6 In vitro degradation tests

Keratin‐PEG hydrogel samples for degradation tests were prepared similarly as described for swelling ratio measurements. Visible light crosslinked hydrogels were lyophilized to record their initial dry weights. Next, the dried samples were allowed to equilibrate in PBS and then incubated at 37 °C with in PBS or Proteinase K solution in PBS (0.5 U/mL) with mild shaking. At different time points, hydrogel samples were lyophilized to record the dry weights. The percentage of remaining mass was the ratio between the final dry weight divided by the initial dry weight.

### 2.7 Mechanical characterization

Keratin‐PEG hydrogel samples for mechanical tests were prepared similarly as described for swelling ratio measurements. Tensile and compressive tests of the keratin‐PEG hydrogels were performed using an Instron mechanical tester (model 5542) with a 10‐N load cell. Dimensions of PMDS molds for hydrogel sample fabrication were 20 × 5 × 1 mm^3^ (cuboid‐shaped) or 8 mm in diameter × 2 mm in thickness (cylindrical) for tensile tests and compressive tests, respectively. Hydrogels were incubated in PBS for 24 hr at 37 °C prior to mechanical testing to reach the equilibrium swelling state, and were then taken out of PBS right before the tests to ensure that the equilibrium swelling state of hydrogel samples did not change. Dimensions of the samples were measured with a digital caliper prior to the tests. For both tests, the strain rate was set as 1 mm/min. The tensile and compressive moduli were determined as the slope of the stress‐strain curve in the linear region corresponding to 0–10% strain.

### 2.8 In vitro cell culture experiments

#### 2.8.1 Cell culture

NIH/3T3 fibroblast cells were cultured in a 5% CO_2_ humidified incubator at 37 °C in Dulbecco's modified eagle medium (Invitrogen) supplemented with 10% fetal bovine serum and 100 U/mL penicillin‐streptomycin. Cells were passaged roughly two times per week and media was changed every other day.

#### 2.8.2 Surface seeding of NIH/3T3 fibroblasts (2D culture)

For 2D cell culture studies, keratin‐PEG hydrogels (10% [w/v] with 0.06 mM Eosin Y) were cast onto 3‐(trimethoxysilyl) propyl methacrylate (TMSPMA, Sigma‐Aldrich) treated glass by exposure to visible light for 60 s. The samples were sterilized with UV light for further 30 min prior to in vitro tests. The glass slides with hydrogels were transferred into a 24‐well plate, seeded with NIH/3T3 cells (20 × 10^3^ cells per sample), and incubated for up to 7 days with media changed every 3 days.

#### 2.8.3 Cell encapsulation of NIH/3T3 fibroblasts (3D culture)

For 3D cell culture studies, cells were collected from the culture flask and resuspended in keratin‐PEG prepolymer solution (10% [w/v] with 0.06 mM Eosin Y) at a concentration of 1 × 10^6^ cells/mL. Cell‐laden keratin‐PEG hydrogels were prepared by exposure to visible light for 60 s covered by TMSPMA treated glass. The hydrogels were then washed thoroughly with media containing Pen/Strep at the concentration of 500 U/mL for sterilization and incubated for up to 7 days with media changed every 3 days.

#### 2.8.4 Cell viability assay

Cell viability was measured with a Live/Dead assay Kit (ThermoFisher) following the manufacturer's manuals. Briefly, the cells were stained with calcein AM (0.5 µL/mL) for live cells and ethidium homodimer‐1 (EthD‐1, 2 µL/mL) for dead cells in PBS. The cells were incubated at 37 °C for 20 min, and thoroughly washed with PBS for three times. The stained cells were then observed using an inverted fluorescence microscope (Nikon TE 2000‐U, Nikon instruments Inc.). The numbers of live and dead cells were counted using the ImageJ software from at least four images taken from different regions of three samples prepared for each experimental condition.

#### 2.8.5 Cell adhesion and spreading

Alexa‐Fluor 594‐conjugated phalloidin (Theremofisher), and 4,6‐diamidino‐2‐phenylindole (DAPI, Sigma‐Aldrich) were used to stain the cells for studying cellular attachment and spreading. Briefly, cell‐seeded hydrogels were fixed in 4% (v/v) paraformaldehyde for 30 min, followed by treatment with a 0.1% (w/v) Triton X‐100 solution in PBS for 20 min to increase permeability, and with a 1% (w/v) bovine serum albumin (BSA) solution in PBS for 1 hr to block nonspecific binding sites. The samples were then incubated in a 1:40 dilution of Alexa‐Fluor 594‐phalloidin in 0.1% (w/v) BSA for 45 min at room temperature to stain the actin cytoskeleton, and then incubated in a 0.1% (w/v) DAPI solution in PBS for 10 min at 37 °C to stain the cell nuclei. After each staining step, the samples were thoroughly washed with PBS before visualizing with the Nikon TE 2000‐U microscope. The ImageJ software was used to count the DAPI stained nuclei and assess the average areas of the cells.

#### 2.8.6 Cell metabolic activity assay

Metabolic activity of cells seeded on or encapsulated in hydrogels was evaluated with the PrestoBlue™ reagent (Invitrogen) following the manufacturer's instructions. The reagent was added at 1× concentration into the culture media and further incubated at 37 °C for 2 hr. Next, 200 µL of the media were transferred to a 96‐well plate to measure absorbance at 570 and 600 nm. Blank media without cells were used as control. Corrected absorbance values were recorded and normalized to the value at day 1. At least three samples were tested at each time point.

### 2.9 Microfabrication of cell‐laden keratin‐PEG hydrogels

#### 2.9.1 Micropatterning using a photomask

Preparation of micropatterned cell‐laden keratin‐PEG hydrogels was similar to the 3D cell culture experiments, except that a photomask was applied on the TMSPMA‐coated cover glass. Light exposure time was found to influence the resolution of hydrogel blocks, which was optimized to 40 s for a 10% (w/v) prepolymer solution with 0.6 mM Eosin Y and 1 × 10^6^ cells/mL. After light crosslinking, the hydrogel was washed with media and incubated for up to 7 days with media changed every 3 days.

#### 2.9.2 Wet spinning

For wet spinning experiments, a solution containing 10% (w/v) keratin‐PEG prepolymer, 1% (w/v) alginate, 0.6 mM Eosin Y, and 1 × 10^6^ cells/mL was prepared and transferred to a syringe. The solution was injected by a syringe pump into 1% (w/v) CaCl_2_ solution to generate the fibers, which were collected and exposed to visible light for 300 s to achieve chemical crosslinking. The fibers were then immersed in 20 mM EDTA for 5 min to remove calcium and alginate before washed by media and transferred in media to incubate for up to 7 days with media changed every 3 days.

### 2.10 Statistical analysis

Data analysis were performed by using one‐way ANOVA followed by Bonferroni's post hoc test in the GraphPad Prism 6.0c software, and presented as mean ± standard deviation of measurements with *p < .05, **p < .01, ***p < .001, and ****p < .0001.

## 3 RESULTS AND DISCUSSION

### 3.1 Preparation of visible light crosslinkable keratin‐PEG hydrogels

Extraction of keratin from human hair using sodium sulfide is the process to break the intermolecular disulfide bonds to make individual protein molecules soluble. During the extraction step, intramolecular disulfide bonds might also be cleaved, thus generating free thiol groups in keratins. However, after the purification step by dialysis and lyophilizing, keratin samples obtained from our extraction procedure showed a very low free thiol content (<0.01 mmol/g) according to the Ellman's assay results, which is similar to previously reported data.^18^ This indicated that the majority of cysteine residues formed intramolecular disulfide bonds. To take advantage of the high reactivity of free thiol groups for the design of photocrosslinkable keratin hydrogels, these intramolecular disulfide bonds should be broken to release the free thiol groups.

**Figure 1:**
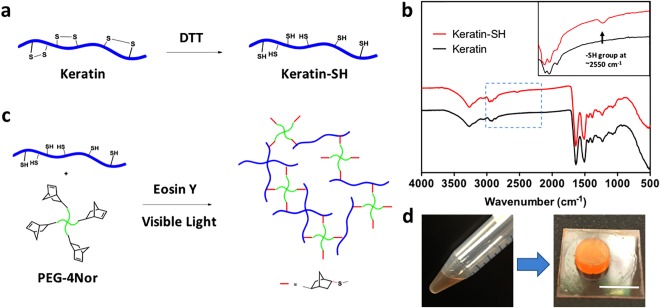
(a) Human keratin extracted from hair was treated with excess amount of DTT at room temperature to break the disulfide bonds and introduce reactive thiol groups. (b) Comparison between the FTIR spectra of Keratin and reduced Keratin‐SH showed an additional peak at about 2550 cm^−1^, which can be assigned to the thiol groups. Inset shows the zoom‐in view of the regions in blue box. (c) Keratin‐SH reacted with a 4‐arm PEG crosslinker bearing four norbornene groups at the presence of Eosin Y as the photoinitiator on visible light activation to form crosslinked hydrogels. (d) Photographs of the prepolymer solution and a visible light crosslinked keratin‐PEG hydrogel. Scale bar: 1 cm

We used DTT, a common reductive reagent known to break disulfide bonds in proteins,^39^ to further react with the extracted keratins to synthesize reduced Keratin‐SH at room temperature (Figure 1a). The free thiol content of the reduced Keratin‐SH samples was determined by the Ellman's assay as ∼0.55 mmol/g, which was significantly higher than the pristine keratin samples. It should also be noted that attempts to purify Keratin‐SH by dialysis against deionized water failed to generate samples with similar thiol content, probably due to the slow oxidation by oxygen during dialysis that brings back the disulfide linkages between cysteine residues. Fourier‐transform infrared spectroscopy (FTIR) was used to identify the difference between pristine keratin and the reduced Keratin‐SH. As shown in Figure 1b, after the reduction an additional weak absorption peak appeared at around 2550 cm^−1^, which can be attributed to the free thiol groups based on a previous report.^40^

After the successful synthesis of Keratin‐SH, we further investigated the experimental conditions to form keratin‐based photocrosslinkable hydrogels. We selected a four‐armed PEG derivative with four norbornene groups at the chain ends as the linker for the crosslinking reaction.^32^, ^33^ Recently, the addition reaction between thiol groups and norbornene groups via the free radical mechanism has attracted increasing attention in the field of biomaterials.^30^, ^31^ Different from the chain photopolymerization of methacrylate monomers that results in heterogeneous crosslinking sites via dynamic chain formation,^25^, ^41^ the thiol‐norbornene reaction took place at a 1:1 stoichiometric ratio to form well‐defined thiol‐ether linkages (Figure 1c). As a result, the network formation is via a tunable step‐growth gelation process.^30^ Under photo‐activation, the reaction kinetics between thiyl radicals and the double bonds in norbornene groups is much faster than most of other side reactions, which renders this reaction a “click”‐type feature.^31^ Importantly, it has been reported that the thiol‐norbornene reaction can be initiated by Eosin Y, a safe visible light photoinitiator that received Food and Drug Administration approval, without the presence of any coinitiator and comonomer, suggesting a convenient, biocompatible and safe strategy to fabricate visible light crosslinkable hydrogels.^27^ Moreover, under the photoinitiated free radical reaction conditions, the norbornene groups cannot polymerize by themselves, thus excluding the probability to form heterogeneous PEG networks that are separated from keratin.^31^ As shown in Figure 1d, exposure under visible light for less than one minute readily turned the prepolymer solution into strong, transparent keratin‐PEG hydrogels, with morphologies that are in sharp contrast to most of the previously reported self‐assembled keratin hydrogels.

### 3.2 Physical characterization of the engineered visible light crosslinked keratin‐PEG hydrogels

The physical properties of the photocrosslinked keratin‐PEG hydrogels were tunable by changing prepolymer formulations and experimental conditions during the crosslinking step. Although it is known that many factors can be explored towards tunable physical properties, in this contribution we chose to keep some factors constant, such as the molecular weight and degree of functionality of the PEG linker. In addition, a stoichiometric feed ratio of the thiol and norbornene groups was expected to result in the highest crosslinking density at the same total prepolymer concentrations.^30^ Specifically, we studied the effects of two parameters, namely, the total prepolymer concentration and photoinitiator concentration, on the physical properties of the keratin‐PEG hydrogels.

**Figure 2:**
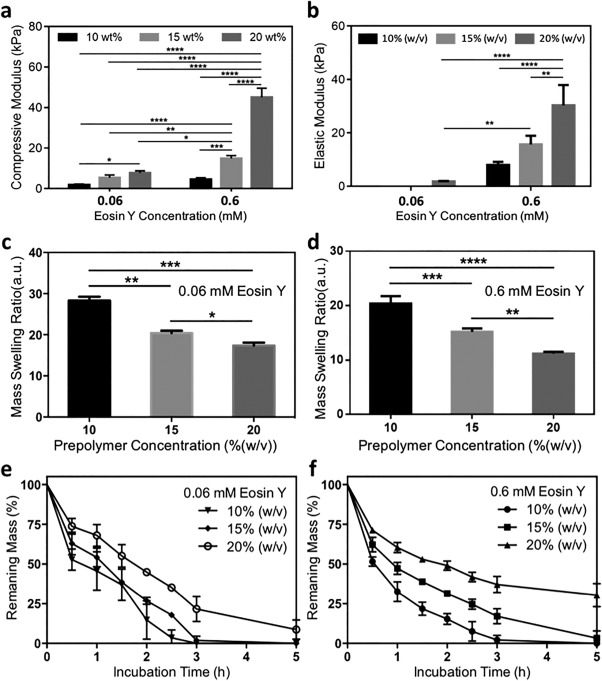
Physical characterization of keratin‐PEG hydrogels. (a) Compressive moduli and (b) elastic moduli of hydrogels of different formulations. (c,d) Equilibrium swelling ratios of keratin‐PEG hydrogels of different formulations using (c) 0.06 mM Eosin Y and (d) 0.6 mM Eosin Y. (e,f) Degradation kinetics profiles of keratin‐PEG hydrogels of different formulations using (e) 0.06 mM Eosin Y and (f) 0.6 mM Eosin Y in 0.5 U/mL Proteinase K solution in PBS at 37°C (*p < .05; **p < .01; ***p < .001; ****p < .0001)

The mechanical characteristics of the keratin‐PEG hydrogels were evaluated by compression and tensile tests using an Instron mechanical machine (Model 5542). As shown in Figure 2a, compressive moduli of the keratin‐PEG hydrogels were found highly dependent on prepolymer and Eosin Y concentrations. At a fixed photoinitiator concentration of 0.06 mmol, the crosslinked samples showed an increase in compressive modulus from 1.9 ± 0.4 kPa for 10% (w/v) hydrogels to 5.4 ± 1.3 kPa and 7.8 ± 1.1 kPa for 15% (w/v) and 20% (w/v) hydrogels, respectively. When Eosin Y concentration was increased to 0.6 mmol, much stiffer hydrogels were formed, and the compressive moduli were 4.6 ± 0.8 kPa, 14.9 ± 1.4 kPa, and 45.0 ± 4.6 kPa for 10, 15, and 20% (w/v) hydrogels, respectively. To our knowledge, a compressive modulus of >40 kPa indicates the strongest keratin‐based hydrogel reported to date.^7^, ^8^, ^24^ Increased compressive moduli at higher prepolymer concentrations might be due to the higher crosslinking density in the resulting hydrogels.^25^, ^38^ The positive correlation between hydrogel mechanical strength and photoinitiator concentration suggested that the tunability of physical properties might rely on the incompleteness of the crosslinking reaction during irradiation.

In tensile tests, keratin‐PEG hydrogels made from prepolymer solutions of a higher photoinitiator concentration (0.6 mM) showed much higher tensile moduli than those made of a lower photoinitiator concentration. Specifically, at a 0.6 mM Eosin Y concentration, the average tensile moduli for 10, 15, and 20% (w/v) hydrogels were 8.0 ± 1.2 kPa, 15.0 ± 3.2 kPa, and 30.3 ± 7.6 kPa, respectively (Figure 2b). When crosslinked at a lower photoinitiator concentration (0.06 mM), the resulting hydrogel samples were not strong enough for the tensile tests. We could only record a low tensile modulus of 1.7 ± 0.4 kPa for the 20% (w/v) keratin‐PEG hydrogels. Overall, through changing the concentrations of prepolymer solution and photoinitiator, hydrogels with a wide range of tensile modulus values could be obtained.

Swelling characteristics are another important physical parameter of hydrogels that might have effects on the mechanical stiffness and diffusion properties of the hydrogel networks. Crosslinking density of the keratin‐PEG hydrogel also significantly influenced the swelling behavior. When reaching the equilibrium swelling state in PBS, it was revealed that in general the determined swelling ratios of different hydrogel samples were inversely proportional to crosslinking density, or more specifically, total prepolymer concentration and photoinitiator concentration in this study. The equilibrium swelling ratios for 10, 15, and 20% (w/v) hydrogels made by using 0.06 mM Eosin Y were calculated as 28.3 ± 1.0, 20.4 ± 0.6, and 17.4 ± 0.7, respectively (Figure 2c). When a higher photoinitiator concentration was used, the crosslinking density of the resulting networks could be increased, thus leading to decreased ability of the network to absorb water molecules. As a result, 10, 15, and 20% (w/v) keratin‐PEG hydrogels made with 0.6 mM Eosin Y showed larger swelling ratios of 20.4 ± 1.4, 15.2 ± 0.6, and 11.2 ± 0.3, respectively (Figure 2g). Tunable swelling characteristics not only change the mechanical properties of hydrogels but also other important features such as diffusion rate of molecules through the hydrogel networks.

Unlike other natural protein‐based biomaterials, it is known that mammalian cells typically do not secrete enzymes for proteolytic degradation of keratin in vivo. Considering the chemical structures of the crosslinked hydrogel network, it is reasoned that degradation of our keratin‐PEG hydrogels could rely on hydrolysis of the PEG crosslinker. In vitro degradation of keratin‐PEG hydrogels by incubation in PBS showed very slow kinetics of mass loss up to 21 days (Supporting Information Figure S1), suggesting potential long‐term use as cell culture matrices for cell therapy. To investigate the dependence of degradation kinetics on hydrogel formulation, we applied Proteinase K, an enzyme extracted from *Tritirachium album* that can digest keratin, to accelerate the degradation process. When treated with 0.5 U/mL Proteinase K, the six tested hydrogel formulations showed varied degradation rates, as shown in Figure 2e,f. Since the degradation of keratin‐PEG hydrogels with Proteinase K was achieved by proteolytic cleavage of the peptide backbone near hydrophobic aliphatic or aromatic amino acid residues, increased protein concentration and crosslinking density within the hydrogel network would slow down the degradation, which qualitatively matched our observed trend. These degradation experiments strongly suggested the formation of a mixed crosslinked network structure between Keratin‐SH and PEG‐4Nor via the coupling of thiol and norbornene groups.

### 3.3 2D cell culture on keratin‐PEG hydrogels

**Figure 3:**
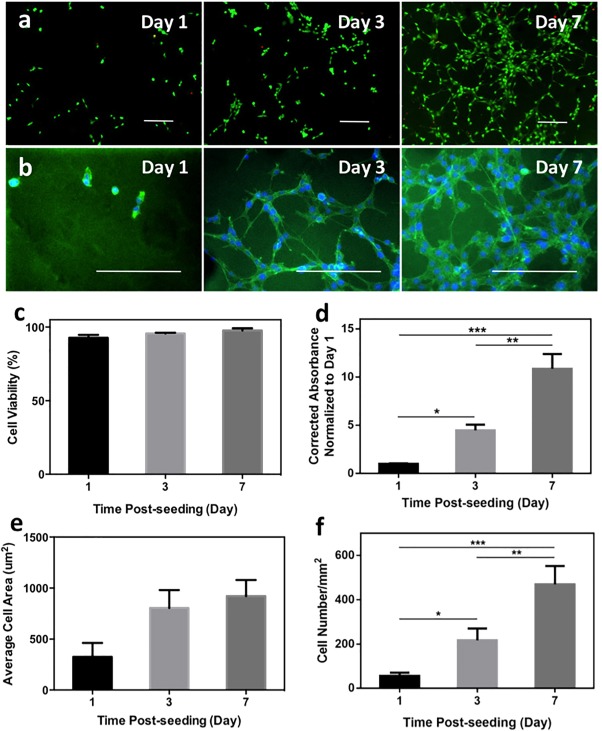
In vitro 2D cell seeding on keratin‐PEG hydrogels. (a) Representative Live/Dead images of stained NIH/3T3 cells seeded on surfaces of keratin‐PEG hydrogels at days 1, 3, and 7 of culture (scale bar: 200 μm). Keratin‐PEG gels were produced from prepolymer solutions of a total 10% (w/v) concentration and 0.06 mM Eosin Y. (b) Representative images of phalloidin/DAPI stained NIH/3T3 cells seeded on hydrogels at days 1, 3, and 7 of culture (scale bar: 200 μm). (c) Quantification of cell viabilities at 1, 3, and 7 days of culture. (d) Measured relative degrees of metabolic activities of NIH/3T3 cells seeded on hydrogels using PrestoBlue assay at days 1, 3, and 7 of culture. (e) Quantification of areas of seeded NIH/3T3 cells obtained from F‐actin/cell nuclei stained images at days 1, 3, and 7 of culture. (f) Cell densities determined by counting the number of DAPI stained nuclei per given surface area of hydrogels at days 1, 3, and 7 of culture (*p < .05, **p < .01, and ***p < .001)

Due to the presence of cell‐adhesive Leu‐Asp‐Val (LDV) motifs in the backbone of keratin,^42^ which can be recognized by α4β1 integrin,^10^, ^16^ it is expected that keratin‐PEG hydrogels can support cellular attachment, although PEG is an inert material. To test this, we selected NIH/3T3 fibroblasts as a commonly used model cell type to investigate the ability of photocrosslinkable keratin‐PEG hydrogels to support 2D cell growth in vitro. We chose 10% (w/v) keratin‐PEG hydrogels formulated by using 0.06 mM Eosin Y in the 2D cell culture study. NIH/3T3 cells were directly seeded on the surface of hydrogels cast on TMSPMA‐treated glass slides and subsequently cultured for 7 days. Cell viability, spreading, and metabolic activity were determined at days 1, 3, and 7 after seeding (Figure 3).

As shown in Figure 3a, cells seeded on keratin‐PEG hydrogels remained viable up to 7 days with high viability (∼90%). F‐actin staining using the Alexa‐Fluor 594‐phallodin clearly showed the elongated cells on hydrogel surfaces at days 3 and 7 (Figure 3b). It is also clear that cells could grow on the hydrogel substrate during in vitro culture. We used the PrestoBlue assay to assess the metabolic activity of the cells seeded on hydrogels and observed significantly increased absorbance values at days 3 and 7 (Figure 3d). Moreover, the cell distribution was relatively uniform on the hydrogel surfaces with certain localized aggregates at day 7, which is different from a previous report that showed highly clustered L929 cells when cultured on self‐assembled keratin hydrogels.^16^ These results suggest a more uniform distribution of the cell‐binding motifs on the keratin‐PEG hydrogel surfaces. From the microscopic images after F‐actin staining, we were able to quantify the average areas of hydrogels covered by the cells at different culture times. The determined average cell areas were found significantly larger at days 3 and 7, compared with that at day 1, confirming that the keratin‐PEG hydrogels can support cell spreading as 2D culture substrates (Figure 3e). After the DAPI staining, it is also feasible to directly count the number of cells by counting the number of stained cell nuclei in the microscopic images. As shown in Figure 3f, the cell density values showed roughly three‐fold and seven‐fold increases at days 3 and 7, respectively, compared to day 1. Overall, these 2D culture experiments using NIH/3T3 fibroblasts indicated that the keratin‐PEG hydrogels could support cell attachment and spreading when used as culture substrates in vitro.

### 3.4 3D cell encapsulation within keratin‐PEG hydrogels

To develop in vitro cell culture models or design synthetic matrix materials for cell delivery, the ability of hydrogels to support 3D cell encapsulation is essential.^43^ Due to the slow curing kinetics of previously reported keratin hydrogels, studies on 3D cell encapsulation using keratin‐based hydrogels have been limited.^18^ Considering the biocompatibility of keratin and the PEG crosslinker and the mild conditions used for photocrosslinking, the visible light crosslinked keratin‐PEG hydrogels could be used to encapsulate cells in vitro. To do this, we used NIH/3T3 fibroblasts as the model to assess cell behaviors within the keratin‐PEG network.

**Figure 4:**
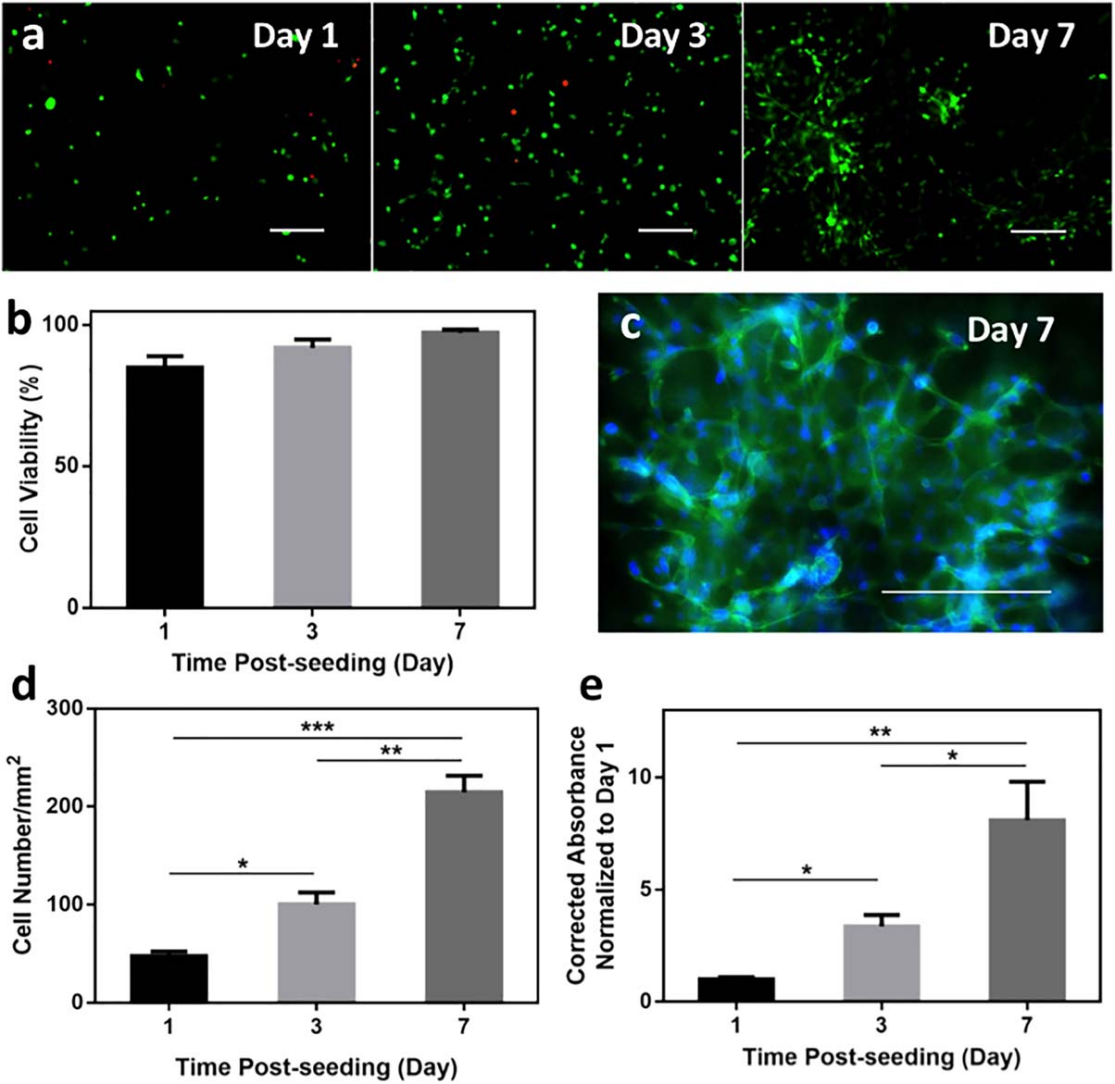
In vitro 3D encapsulation of NIH/3T3 cells in keratin‐PEG hydrogels. (a) Representative Live/Dead images of NIH/3T3 cells encapsulated in keratin‐PEG hydrogels at days 1, 3, and 7 of culture (scale bar: 200 μm). Keratin‐PEG hydrogels were produced from prepolymer solutions of a total 10% (w/v) concentration and 0.06 mM Eosin Y. (b) Quantification of cell viabilities at 1, 3, and 7 days of culture. (c) Representative image of phalloidin/DAPI stained NIH/3T3 cells encapsulated in hydrogels at day 7 of culture (scale bar: 200 μm). (d) Cell densities determined by counting the number of DAPI stained nuclei per given area at days 1, 3, and 7 of culture. (e) Measured relative degrees of metabolic activities of seeded NIH/3T3 cells using PrestoBlue assay at days 1, 3, and 7 of culture (*p < .05, **p < .01, ***p < .001)

As shown in Figure 4a, Live/Dead staining of the cell‐laden hydrogels indicated that the embedded cells remained alive up to day 7 of culture with high viability (>85%) (Figure 4b), which suggested that both the materials and the photocrosslinking conditions were compatible to fibroblasts. The capability to develop photocrosslinkable cell‐laden keratin hydrogels would expand the utilization of keratin‐based biomaterials in various tissue engineering applications, due to the unique features of the photocrosslinking mechanism, which typically allows rapid hydrogel formation and precise spatiotemporal control over hydrogel properties. When embedded in the keratin‐PEG matrices, the fibroblasts showed round morphology at day 1 and started to elongate at day 3. At day 7, apparent cell elongation and proliferation could be observed, with the formation of cell clusters at certain regions of the hydrogel constructs. Although interfered by the background fluorescence from the keratin material, we observed the spreading cell morphology after staining for F‐actin at day 7 (Figure 4c). Since we fabricated very thin cell‐laden hydrogels (∼150 μm in thickness) with uniform cell distribution, we were able to quantify the cell numbers by counting the cell nuclei after DAPI staining, which showed roughly two‐fold and four‐fold increases at days 3 and 7, respectively, compared to day 1 (Figure 4d). Conversely, cell metabolic activity can be similarly determined using the PrestoBlue assay in the thin cell‐laden hydrogel samples, assuming that diffusion is not a severe problem for the assay reagent. As shown in Figure 4e, when normalized to the value at day 1, the absorbance values increased by a factor of roughly 3.5 and 7.5 at days 3 and 7, respectively.

From these in vitro 3D encapsulation experiments, it is suggested that the photocrosslinkable keratin‐PEG hydrogels provide an additional choice as cytocompatible model culture systems. In addition, due to the presence of cell‐binding LDV peptide sequences, our keratin‐based hydrogels showed the ability to support cell spreading in the matrices. It should be noted that the spreading morphology of encapsulated NIH/3T3 fibroblasts at day 7 depends on the crosslinking density of the hydrogel matrix and can be observed in keratin‐PEG hydrogels with low prepolymer (10% [w/v]) and Eosin Y (0.06 mM) concentrations. When Since it is believed that fibroblasts cannot produce enzymes to degrade keratins,^18^ the matrix remodeling might take place via the hydrolytic cleavage mechanism. When encapsulated in hydrogels with higher crosslinking density, the spreading of NIH/3T3 cells might be slowed down.

### 3.5 Microfabrication of keratin‐PEG hydrogels

The generation of organized assemblies of cells is a critical factor to resemble the complex microstructures of the native tissues. Microfabrication technologies such as micropatterning or wet spinning can be used to control cell orientation and organization within 3D hydrogels in order to engineer biomimetic 3D tissue constructs.^34^, ^44^ Therefore, we investigated the capability of our keratin‐based hydrogel to be integrated with microscale technologies for the engineering of tissue constructs with controlled architectures. The photocrosslinkable keratin‐PEG hydrogel platform indicates various possibilities to combine with different microfabrication technologies due to the versatility of the photocrosslinking mechanism.^35^ Microfabricated hydrogels are useful in vitro cell culture models to study cell‐biomaterial or cell‐cell interactions.^45^ Here, we select two representative microfabrication techniques, namely, photopatterning^34^, ^35^ and wet spinning,^37^ as the examples to demonstrate the potentials of this keratin hydrogel system in bioengineering applications.

**Figure 5:**
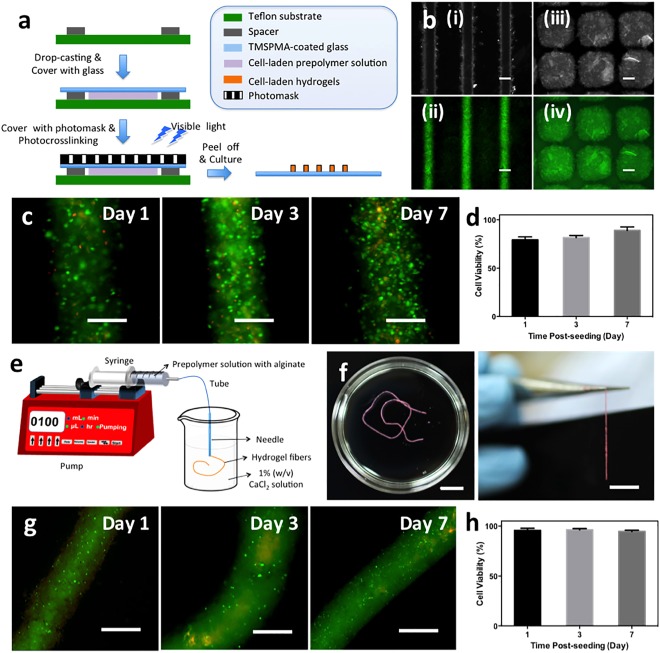
Microfabricated keratin‐PEG hydrogels. (a) Schematic illustration of the photopatterning process to generate keratin‐PEG hydrogel blocks that duplicate the patterns of the photomask. (b) Representative microscopic images of microfabricated (i, ii) hydrogel lines and (iii, iv) square blocks (scale bar: 200 μm). (c) Representative Live/Dead images of stained NIH/3T3 cells encapsulated in keratin‐PEG hydrogel lines at days 1, 3, and 7 of culture (scale bar: 200 μm). Keratin‐PEG gels were produced from prepolymer solutions of a total 10% (w/v) concentration and 0.6 mM Eosin Y. (d) Quantification of cell viabilities encapsulated in micropatterned hydrogels at days 1, 3, and 7 of culture. (e) Schematic illustration of the wet spinning process to generate hydrogel microfibers. (f) Photographs of the hydrogel fibers after photocrosslinking (scale bar: 1 cm). (g) Representative Live/Dead images of stained NIH/3T3 cells encapsulated in keratin‐PEG fibers at days 1, 3, and 7 of culture (scale bar: 500 μm). (h) Quantification of cell viabilities in fabricated fibers at days 1, 3, and 7 of culture

Photopatterned cell‐laden keratin‐PEG hydrogels were readily fabricated using photomasks with different patterns and feature sizes (Figure 5a,b). By optimizing the photocrosslinking conditions, hydrogel lines with widths below 200 µm could be obtained with high shape resolution. In our previous studies, it has been shown that the line width could be tuned by using different photomasks, varied light exposure time, or adjusting the distance between photomask and the prepolymer solution.^35^ Moreover, using photomasks with different shapes of patterns, square blocks of keratin‐PEG hydrogels were also obtained (Figure 5b, iii,iv). To demonstrate that the photopatterning process is compatible to cells, viability of cells embedded in the patterned hydrogel matrices were assessed with the Live/Dead assay (Figure 5c), which showed that the cells remained viable for at least 7 days of culture (80% or higher viability) (Figure 5d). However, since the optimized hydrogel formulation for photopatterning contains a higher Eosin Y concentration (0.6 mM), which resulted in hydrogels with higher crosslinking density and thus limited the spreading of cells at day 7 of culture.

Cell‐laden hydrogel microfibers are promising building blocks to combine with textile techniques to fabricate tissue constructs.^36^, ^37^ To demonstrate the feasibility of our hydrogels to prepare cell‐laden microfibers, we followed a wet spinning protocol recently developed by our group, which was based on the capability that alginate could rapidly form physical crosslinking with divalent cations such as calcium ions.^46^ To this end, the keratin‐PEG prepolymer solution was blended with alginate to form a composite mixture to encapsulate with cells. The cell‐laden composite solution was directly injected through a needle into a CaCl_2_ solution (Figure 5e), which resulted in the formation of continuous and uniform hydrogel fibers crosslinked by alginate/Ca^2+^ complexation with up to at least tens of centimeters in length (Figure 5f). After a second step of photocrosslinking by exposure to visible light, the calcium ions could be washed away by ethylenediaminetetraacetic acid disodium salt (EDTA), a chelator to calcium ions. Importantly, we have demonstrated that the physical crosslinking of alginate with calcium and the removal by EDTA solutions were cytocompatible to encapsulated cells, making it a unique method towards cell‐laden fibrous tissue constructs.^36^, ^37^ As shown in Figure 5g,h, although showing no cell spreading due to the highly crosslinked hydrogel matrix, the embedded cells remained highly viable through the wet spinning process and subsequent in vitro culture up to 7 days. It should be noted that although the fabrication of microfibers from keratin‐PEG hydrogels is aided by the physical crosslinking of alginate and Ca^2+^ ions, the second chemical photocrosslinking of Keratin‐SH and PEG‐4Nor contributed to the long‐term stability after the removal of Ca^2+^ ions. Using these two illustrations of microfabrication of keratin‐PEG hydrogels, we expect that this visible light crosslinkable hydrogel system finds widespread applications to engineering cell‐laden tissue constructs with various architectures and compositions using a human protein as the basic material.

## 4 CONCLUSION

In this contribution, we reported the preparation and characterization of a photocrosslinkable hydrogel based on human hair keratin and a PEG crosslinker, which showed rapid crosslinking, highly tunable physical properties, and high cytocompatibility. By enriching free thiol groups on keratin and introducing norbornene groups to the PEG crosslinker, photocrosslinking was achieved by the “click‐type” thiol‐norbornene addition reaction upon visible light exposure using Eosin Y as the photoinitiator. Using NIH/3T3 fibroblasts as the model cell type, we demonstrated that the engineered keratin‐PEG hydrogel system could be used as both 2D and 3D in vitro cell culture substrates, which supported cell adhesion and spreading due to the presence of cell‐binding peptide motifs. Moreover, taking advantage of the photocrosslinking mechanism, we showed that this hydrogel system was suitable for microfabrication techniques, such as photopatterning and wet spinning, to produce cell‐laden hydrogel constructs with different shapes. Our future work will focus on developing a large‐scale 3D construct using keratin‐based hydrogel through engineering vasculature and perfusion system, combining with 3D microfabrication technologies developed previously in our lab.^46^, ^47^ We believe that the engineered photocrosslinkable hydrogel based on extracted human hair keratin might find widespread applications in tissue engineering.

## Supporting information

Additional Supporting Information may be found online in the supporting information tab for this article.

Supporting Figure

## References

[1] Kundu B , Rajkhowa R , Kundu SC , Wang X. Silk fibroin biomaterials for tissue regenerations. Adv Drug Deliv Rev. 2013;65:457–470. 23137786 10.1016/j.addr.2012.09.043

[2] Altman GH , Diaz F , Jakuba C , et al. Silk‐based biomaterials. Biomaterials. 2003;24:401–416. 12423595 10.1016/s0142-9612(02)00353-8

[3] Chevallay B , Herbage D. Collagen‐based biomaterials as 3D scaffold for cell cultures: applications for tissue engineering and gene therapy. Med Biol Eng Comput. 2000;38:211–218. 10829416 10.1007/BF02344779

[4] Ramshaw JAM , Peng YY , Glattauer V , Werkmeister JA. Collagens as biomaterials. J Mater Sci: Mater Med. 2008;20:3–8. 10.1007/s10856-008-3415-4 18379858

[5] Wise SG , Mithieux SM , Weiss AS. Engineered tropoelastin and elastin‐based biomaterials. In: Alexander M, ed. Advances in Protein Chemistry and Structural Biology. Vol.78 Academic Press, 2009:1–24. 20663482 10.1016/S1876-1623(08)78001-5

[6] Annabi N , Mithieux SM , Zorlutuna P , et al. Engineered cell‐laden human protein‐based elastomer. Biomaterials. 2013;34:5496–5505. 23639533 10.1016/j.biomaterials.2013.03.076 PMC3702175

[7] Lee H , Noh K , Lee SC , et al. Human hair keratin and its‐based biomaterials for biomedical applications. Tissue Eng Regen Med. 2014;11:255–265.

[8] Rouse JG , Van Dyke ME. A review of keratin‐based biomaterials for biomedical applications. Materials 2010;3:999.

[9] Kitahara T , Ogawa H. The extraction and characterization of human nail keratin. J Dermatol Sci. 1991;2:402–406. 1726055 10.1016/0923-1811(91)90003-g

[10] Reichl S. Films based on human hair keratin as substrates for cell culture and tissue engineering. Biomaterials. 2009;30:6854–6866. 19783297 10.1016/j.biomaterials.2009.08.051

[11] Reichl S , Borrelli M , Geerling G. Keratin films for ocular surface reconstruction. Biomaterials. 2011;32:3375–3386. 21316757 10.1016/j.biomaterials.2011.01.052

[12] Borrelli M , Joepen N , Reichl S , et al. Keratin films for ocular surface reconstruction: evaluation of biocompatibility in an in‐vivo model. Biomaterials. 2015;42:112–120. 25542799 10.1016/j.biomaterials.2014.11.038

[13] Katoh K , Tanabe T , Yamauchi K. Novel approach to fabricate keratin sponge scaffolds with controlled pore size and porosity. Biomaterials. 2004;25:4255–4262. 15046915 10.1016/j.biomaterials.2003.11.018

[14] Tachibana A , Kaneko S , Tanabe T , Yamauchi K. Rapid fabrication of keratin–hydroxyapatite hybrid sponges toward osteoblast cultivation and differentiation. Biomaterials. 2005;26:297–302. 15262471 10.1016/j.biomaterials.2004.02.032

[15] Hartrianti P , Nguyen LTH , Johanes J , et al. Fabrication and characterization of a novel crosslinked human keratin‐alginate sponge. J Tissue Eng Regen Med. 2016;4:524–531. doi:10.1002/term.2159 10.1002/term.2159 27109145

[16] Wang S , Taraballi F , Tan LP , Ng KW. Human keratin hydrogels support fibroblast attachment and proliferation in vitro. Cell Tissue Res. 2012;347:795–802. 22287039 10.1007/s00441-011-1295-2

[17] Silva R , Singh R , Sarker B , et al. Hybrid hydrogels based on keratin and alginate for tissue engineering. J Mater Chem B. 2014;2:5441–5451. 10.1039/c4tb00776j 32261764

[18] Wang S , Wang Z , Foo S , et al. Culturing fibroblasts in 3D human hair keratin hydrogels. ACS Appl Mater Interfaces. 2015;7:5187–5198. 25690726 10.1021/acsami.5b00854

[19] Li Q , Zhu L , Liu R , et al. Biological stimuli responsive drug carriers based on keratin for triggerable drug delivery. J Mater Chem. 2012;22:19964–19973.

[20] Nakata R , Osumi Y , Miyagawa S , Tachibana A , Tanabe T. Preparation of keratin and chemically modified keratin hydrogels and their evaluation as cell substrate with drug releasing ability. J Biosci Bioeng. 2015;120:111–116. 25561327 10.1016/j.jbiosc.2014.12.005

[21] Shen D , Wang X , Zhang L , et al. The amelioration of cardiac dysfunction after myocardial infarction by the injection of keratin biomaterials derived from human hair. Biomaterials. 2011;32:9290–9299. 21885119 10.1016/j.biomaterials.2011.08.057

[22] Hill PS , Apel P , Barnwell J , et al. Repair of peripheral nerve defects in rabbits using keratin hydrogel scaffolds. Tissue Eng Part A. 2011;17:1499–1505. 21275820 10.1089/ten.TEA.2010.0184

[23] Pace LA , Plate JF , Smith TL , Van Dyke ME. The effect of human hair keratin hydrogel on early cellular response to sciatic nerve injury in a rat model. Biomaterials. 2013;34:5907–5914. 23680369 10.1016/j.biomaterials.2013.04.024

[24] Hill P , Brantley H , Van Dyke M. Some properties of keratin biomaterials: kerateines. Biomaterials. 2010;31:585–593. 19822360 10.1016/j.biomaterials.2009.09.076

[25] Nichol JW , Kosh ST , Bae H , et al. Cell‐laden microengineered gelatin methacrylate hydrogels. Biomaterials. 2010;31:5536–5544. 20417964 10.1016/j.biomaterials.2010.03.064 PMC2878615

[26] Ifkovits JL , Burdick JA. Review: photopolymerizable and degradable biomaterials for tissue engineering applications. Tissue Eng. 2007;13:2369–2385. 17658993 10.1089/ten.2007.0093

[27] Shih H , Lin C‐C. Visible‐light‐mediated thiol‐Ene hydrogelation using eosin‐Y as the only photoinitiator. Macromol Rapid Commun. 2013;34:269–273. 23386583 10.1002/marc.201200605

[28] Hoyle CE , Bowman CN. Thiol–Ene click chemistry. Angew Chem Int Ed Engl. 2010;49:1540–1573. 20166107 10.1002/anie.200903924

[29] Uygun M , Tasdelen MA , Yagci Y. Influence of type of initiation on Thiol–Ene “Click” chemistry. Macromol Chem Phys. 2010;211:103–110.

[30] Fairbanks BD , Schwartz MP , Halevi AE , et al. A versatile synthetic extracellular matrix mimic via thiol‐norbornene photopolymerization. Adv Mater. 2009;21:5005–5010. 25377720 10.1002/adma.200901808 PMC4226179

[31] Lin C‐C , Ki CS , Shih H. Thiol‐norbornene photo‐click hydrogels for tissue engineering applications. J Appl Polym Sci. 2015;132:41563. 25558088 10.1002/app.41563 PMC4280501

[32] Aimetti AA , Machen AJ , Anseth KS. Poly(ethylene glycol) hydrogels formed by thiol‐ene photopolymerization for enzyme‐responsive protein delivery. Biomaterials. 2009;30:6048–6054. 19674784 10.1016/j.biomaterials.2009.07.043 PMC2761537

[33] Anderson SB , Lin C‐C , Kuntzler DV , Anseth KS. The performance of human mesenchymal stem cells encapsulated in cell‐degradable polymer‐peptide hydrogels. Biomaterials. 2011;32:3564–3574. 21334063 10.1016/j.biomaterials.2011.01.064 PMC3085912

[34] Aubin H , Nichol JW , Hutson CB , et al. Directed 3D cell alignment and elongation in microengineered hydrogels. Biomaterials. 2010;31:6941–6951. 20638973 10.1016/j.biomaterials.2010.05.056 PMC2908986

[35] Nikkhah M , Eshak N , Zorlutuna P , et al. Directed endothelial cell morphogenesis in micropatterned gelatin methacrylate hydrogels. Biomaterials. 2012;33:9009–9018. 23018132 10.1016/j.biomaterials.2012.08.068 PMC3643201

[36] Akbari M , Tamayol A , Laforte V , et al. Composite living fibers for creating tissue constructs using textile techniques. Adv Funct Mater. 2014;24:4060–4067. 25411576 10.1002/adfm.201303655 PMC4233137

[37] Tamayol A , Najafabadi AH , Aliakbarian B , et al. Hydrogel templates for rapid manufacturing of bioactive fibers and 3D constructs. Adv Healthcare Mater. 2015;4:2146–2153. 10.1002/adhm.201500492 PMC4767703 26304467

[38] Zhang Y‐N , Avery RK , Vallmajo‐Martin Q , et al. A highly elastic and rapidly crosslinkable elastin‐like polypeptide‐based hydrogel for biomedical applications. Adv Funct Mater. 2015;25:4814–4826. 26523134 10.1002/adfm.201501489 PMC4623594

[39] Singh R , Whitesides GM. Reagents for rapid reduction of native disulfide bonds in proteins. Bioorg Chem. 1994;22:109–115.

[40] Yu D , Cai JY , Church JS , Wang L. Modifying surface resistivity and liquid moisture management property of keratin fibers through thiol–Ene click reactions. ACS Appl Mater Interfaces. 2014;6:1236–1242. 24367993 10.1021/am405060x

[41] Yue K , Trujillo‐de Santiago G , Alvarez MM , et al. Synthesis, properties, and biomedical applications of gelatin methacryloyl (GelMA) hydrogels. Biomaterials. 2015;73:254–271. 26414409 10.1016/j.biomaterials.2015.08.045 PMC4610009

[42] Tselepis VH , Green LJ , Humphries MJ. An RGD to LDV motif conversion within the Disintegrin Kistrin generates an integrin antagonist that retains potency but exhibits altered receptor specificity: evidence for a functional equivalence of acidic integrin‐binding motifs. J Biol Chem. 1997;272:21341–21348. 9261147 10.1074/jbc.272.34.21341

[43] Tay CY , Muthu MS , Chia SL , et al. Reality check for nanomaterial‐mediated therapy with 3D biomimetic culture systems. Adv Funct Mater. 2016;26:4046–4065.

[44] Zorlutuna P , Annabi N , Camci‐Unal G , et al. Microfabricated biomaterials for engineering 3D tissues. Adv Mater Weinheim. 2012;24:1782–1804. 22410857 10.1002/adma.201104631 PMC3432416

[45] Annabi N , Tamayol A , Uquillas JA , et al. 25th anniversary article: Rational design and applications of hydrogels in regenerative medicine. Adv Mater Weinheim. 2014;26:85–124. 24741694 10.1002/adma.201303233 PMC3925010

[46] Jia W , Gungor‐Ozkerim PS , Zhang YS , et al. Direct 3D bioprinting of perfusable vascular constructs using a blend bioink. Biomaterials. 2016;106:58–68. 27552316 10.1016/j.biomaterials.2016.07.038 PMC5300870

[47] Byambaa B , Sakr MA , Seo J , et al. Bioprinted osteogenic and vasculogenic patterns for engineering 3D bone tissue. Adv Healthcare Mater. 2017;6:1700015. doi:10.1002/adhm.201700015 10.1002/adhm.201700015 PMC11034848 28524375

