# Supplementary material for: Visible light crosslinkable human hair keratin hydrogels
Source: Bioeng Transl Med. 2018 Jan 19;3(1):37–48. doi: 10.1002/btm2.10077 (PMC5773942; doi:10.1002/btm2.10077)
Supplement: Supplementary file 1 — Supporting Figure [file BTM2-3-37-s001.docx]

Supporting Information

*for*

Visible Light Crosslinkable Human Hair Keratin Hydrogels

*Kan Yue,^a,b,#^ Yanhui Liu,**^a,b,c,#^ Batzaya Byambaa,^a,b,#^ Vaishali Singh,^d^ Wanjun Liu,^a,b^ Xiuyu Li,^a,b,e^ Yunxia Sun,^a,b,f^ Shrike Yu Zhang,^a,b,g^ Ali Tamayol,^a,b,g^ Peihua Zhang,^c^ Kee Woei Ng,^d,*^ Nasim Annabi,^a,b,g,h,*^ Ali Khademhosseini^a,b,g,i,j,*^*

^a^ Biomaterials Innovation Research Center, Division of Biomedical Engineering, Department of Medicine, Brigham and Women's Hospital, Harvard Medical School, Boston, MA, USA

^b^ Harvard-MIT Division of Health Sciences and Technology, Massachusetts Institute of Technology, Cambridge, MA, USA

^c^ College of Textiles, Donghua University, Shanghai, 201620, China

^d^ School of Materials Science and Engineering, Nanyang Technological University, N4.1, 50 Nanyang Avenue, Singapore 639798, Singapore

^e^ Research Center for Analysis and Measurement, Hebei Normal University, Shijiazhuang 050024, Hebei, China

^f^ Department of Chemistry and Key Laboratory of Biomedical Polymers, Ministry of Education, Wuhan University, Wuhan 430072, China

^g^ Wyss Institute for Biologically Inspired Engineering, Harvard University, Boston, MA, USA

^h^ Department of Chemical Engineering, Northeastern University, Boston, MA, 02115-5000, USA

^i^ Department of Bioindustrial Technologies, College of Animal Bioscience and Technology, Konkuk University, Hwayang-dong, Gwangjin-gu, Seoul 143-701, Republic of Korea

^j^ Department of Physics, King Abdulaziz University, Jeddah 21569, Saudi Arabia

^#^ These authors contributed equally to this work.

[*] Corresponding author: Ali Khademhosseini (Email: [alik@bwh.harvard.edu)](mailto:alik@bwh.harvard.edu)); Nasim Annabi (Email: [n.annabi@neu.edu](mailto:n.annabi@neu.edu)); and Kee Woei Ng (E-mail: [kwng@ntu.edu.sg)](mailto:kwng@ntu.edu.sg)).


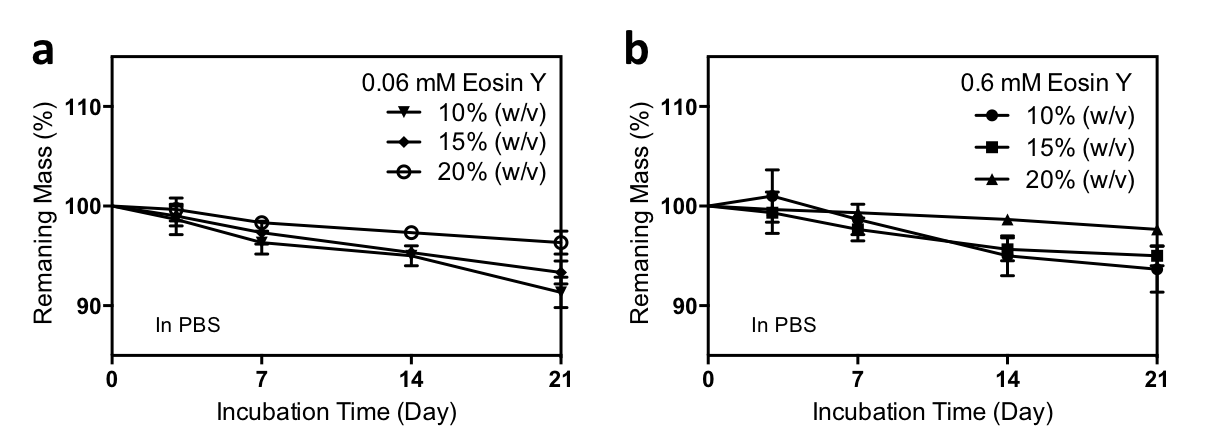


**Figure S1.** Degradation kinetics profiles of keratin-PEG hydrogels of different formulations using **(a)** 0.06 mM Eosin Y and **(b)** 0.6 mM Eosin Y in PBS at 37 °C
